# Purging the HIV-1 reservoir through the disruption of the PD-1 pathway

**DOI:** 10.1186/1758-2652-13-S3-O15

**Published:** 2010-11-04

**Authors:** S DaFonseca, N Chomont, M El Far, R Boulassel, J Routy, R Sékaly

**Affiliations:** 1 Vaccine and Gene Therapy Institute, 11350 SW Village Parkway, Port St. Lucie, Florida 34987, USA; 2 Laboratoire d’Immunologie, Centre de Recherche du Centre Hospitalier de l’Université de Montréal (CR-CHUM) Saint-Luc, 264 René Lévesque Est, Montréal, Québec H2X1P1, Canada; 3 Laboratoire d’Immunologie, Département de Microbiologie et d’Immunologie, Université de Montréal, Québec, Canada; 4 INSERM U743, CR-CHUM, Université de Montréal, 264 René Lévesque Est, Montréal, Québec H2X1P1, Canada; 5 Immunodeficiency Service and Division of Hematology, Royal Victoria Hospital, McGill University Health Centre (MUHC), McGill University, 687 Pine Avenue West, Montréal, Québec H3A 1A1, Canada; 6 Department of Microbiology and Immunology, 3775 University Street, McGill University, Montréal, Québec H3A2B4, Canada

## Background

The main obstacle to HIV-1 eradication is a small pool of T_CM_ (central memory) and T_TM_ (transitional memory) latently infected CD4+ T cells that persist in patients receiving HAART. The mechanisms implicated in the establishment and persistence of the HIV reservoir are still unknown. The PD-1 receptor is expressed by CD4+ T cells from HIV-infected patients, and efficiently inhibits T cell proliferation. Here, we investigate a possible role for this receptor in the establishment and maintenance of a cellular reservoir for HIV.

## Methods

PBMCs were obtained by leukapheresis from HAART-naïve, chronically HIV-1-infected subjects and were subjected to total CD4+ T cells negative selection. Viral production was induced through TCR triggering (CD3/CD28) with or without co-triggering of the PD-1 pathway with an Ig-PD-L1 chimera. Cell culture supernatants were serially harvested; viral release was quantified by QRT-PCR or p24 ELISA. The frequency of CD4+ T cells harbouring HIV DNA was determined by Q-PCR.

## Results

Cell sorting and Q-PCR experiments showed that PD-1^high^ cells from viremic donors preferentially harbour HIV-1 integrated DNA when compared with their PD-1^low^ counterparts, indicating that these cells constitute a preferential reservoir for the virus. Triggering of the PD-1 pathway inhibits 50% of HIV-1 production in primary CD4+ T cells at day 1 and up to 95% at day 3. Importantly, this inhibition was restricted to PD-1^high^ cells, demonstrating the specificity of this mechanism. Moreover, we observed that the disruption of the PD-1/PD-L1 interaction enhances the spontaneous release of HIV-1 virions by CD4+ T cells.

## Conclusions

Our results suggest that: (1) the PD-1 receptor can be used as a specific marker to target the HIV-1 reservoir; (2) viral production is inhibited after triggering of the PD-1 receptor; and (3) viral production was enhanced after blocking of this inhibitory pathway. Altogether, our results demonstrate a crucial role for PD-1 in the establishment.

